# The mitochondrial genome of *Diaphanosoma* *excisum* Sars, 1885 (Crustacea: Branchiopoda: Cladocera) from Hainan Island, China

**DOI:** 10.1080/23802359.2021.1907252

**Published:** 2021-03-28

**Authors:** Jiaying Pan, Ping Liu, Franja Pajk, Henri J. Dumont, Bo-Ping Han

**Affiliations:** a Department of Ecology, Jinan University, Guangzhou, PR China; b Department of Biology, University of Gent, Gent, Belgium

**Keywords:** *Diaphanosoma excisum*, freshwater Cladocera, mitogenome, phylogenetic analysis

## Abstract

*Diaphanosoma excisum* is the only Cladoceran in tropical freshwaters and parapatrically occurs with *Diaphanosoma dubium* in the transition between the tropics and subtropics. Here, we present the complete mitochondrial genome (MG) determined by next-generation sequencing and offer a phylogenetic analysis of *D. excisum*. The MG of *D. excisum* is 17,615 bp in size, including 13 protein-coding genes (PCGs), 2 ribosomal RNA, 23 tRNA, and 2 putative control regions. The MG has a biased A + T of 65.34% for base composition. Compared to *D. dubium*, the MG of *D. excisum* has one more tRNA-Met, one unknown extra putative control region and is different in the arrangement of its tRNAs. The MG sequence and tRNA order provide valuable molecular data for understanding the phylogeny and speciation of *Diaphanosoma*.

*Diaphanosoma*, the ‘tropical *Daphnia*’, is common and ubiquitous in the tropics and subtropics (Dumont 1994; Sarma et al. 2005; Dumont et al. 2021). Among *Diaphanosoma* species, *Diaphanosoma excisum* and *Diaphanosoma dubium* are two of the most common and dominant species. They are parapatrically distributed in warmer waters and rarely coexist in the transition zone between the tropics and subtropics (Korovchinsky et al. 2017; Liu et al. 2018; Pajk et al. 2018). In the tropics, *D. excisum* is frequently the only Cladoceran present (Kotov et al. 2013). In contrast to *D. dubium*, which is widespread in subtropical waters and with a dominance in China, *D. excisum* is restricted to the coastal islands of the southern part of the country (Chen et al. 2011). Pajk et al. (2018) measured the life history traits of clones from 16 populations of *D. dubium* and *D. excisum* under a broad temperature range from 10 °C to 40 °C and showed that *D. excisum* had a narrower thermal performance curve (TPC) and a higher optimum temperature than the subtropical *D. dubium*, but failed to reproduce at ≤15 °C. Stable thermal niche difference is considered to play a critical role in shaping *Diaphanosoma* species range. To reveal the potential mechanism underlying niche divergence of the two congeners, there is a need to analyze not only ecological, but also genetic information. Here, we sequenced and annotated the mitochondrial genome (MG) of *D. excisum* and compared it to the published MG of *D. dubium* (Liu et al. 2017).

Living animals were collected from Donghu lake (110.35°E, 20.04°N) in Hainan Island, China, and mass-cultured in a small aquarium. The specimens (accession number COZOOP02002B, Ningning Liu (osss@jnu.edu.cn)) and their extracted DNA were preserved at −20 °C in the Aquatic Collection of Institute of Hydrobiology, Jinan University, Guangzhou, China. Thousands of individuals were collected and preserved in −80 °C. Genomic DNA was extracted using the TIANamp Marine Animals DNA Kit (TIANGEN BIOTECH CO., LTD (Beijing, China) and sequenced using a next-generation method on Illumina platform HiSeq 2500. The assembly and annotation procedure followed Xu et al. (2018) with the COI sequence of *D. excisum* as the seed. The assembled sequence was annotated in MITOS WebServer (Bernt et al. 2013). Base depth was qualified with BamDeal (https://github.com/BGI-shenzhen/BamDeal). Transfer RNA genes were conformed with tRNAscan-SE version 2 (Lowe and Eddy 1997). To verify the putative control regions, we re-sequenced individual animals of *D. excisum* with four pairs of primers, i.e. DE1F: ggcgtgatgagatggtgaatta and DE1R: ggctgcaacaaacccataaac, DE2F: gaacggcaagacgagagaaa and DE2R: cgttgggtatcacgacagtaaa, DE3F: tcgtacgctctcgtacctatac and DE3R: gccgactttggcttcatcta, DE4F: gcgacctcgatgttggatta and DE4R: cgcatagagacacatgggtatag.

The complete MG size of *D. excisum* (GenBank accession number: MW476927) was 17,615 bp. It included 13 protein-coding genes (PCGs), 2 rRNAs, 23 tRNAs, and 2 putative control regions. The nucleotide composition was A + T biased with GC content of 34.66%. Most PCGs are initiated by a typical ‘ATN’ codon, while COX1 uses ‘TTG’ as the start codon. The tRNA genes have lengths ranging from 63 to 72 bp, and they are fold into a typical cloverleaf structure. The two putative control regions were 1143 and 1215 bp in length, respectively. However, one more tRNA-Met was detected in *D. excisum*, which was uncommon for invertebrate. The same phenomenon was also found in the MG (GenBank accession number: MT356995) of another *Diaphanosoma* species. The arrangement of tRNA was different for the two species, but no shift of PCGs was discovered. Besides, one more putative control region was detected in *D. excisum*.

To explore the phylogenetic relationships of *D. excisum* and *D. dubium* and seven other species from three families in Brachiopoda which have complete MGs, a phylogenetic tree was obtained using Bayesian inference (BI) analysis based on entire PCGs sequences and two rRNA sequences. The Bayesian analysis was performed using MrBayes version 3.1.2 (Ronquist and Huelsenbeck 2003) with the GTR + G+I model of nucleotide substitution. The MGs of the two *Diaphanosoma* species have a similar A + T composition (65.34% for *D. excisum* and 65.6% for *D. dubium*). The phylogenetic tree shows that *D. excisum* was fully resolved in a clade with *D. dubium* in the Sididae (Figure 1). The Sididae (Ctenopda) was phylogenetically closer to the anomopod Daphniidae, but divergent from Triopsidae (order Notostraca) and Artemiidae (order Anostraca).

**Figure 1.**
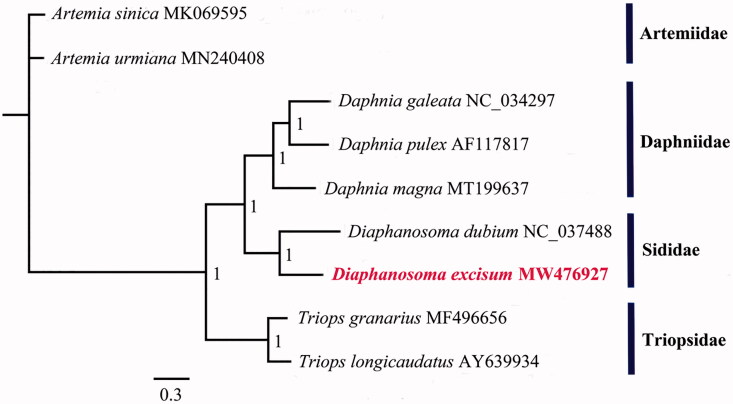
Phylogenetic estimate of the position of *D. excisum* and eight other species of Branchiopod Crustaceans. The numbers at the nodes are posterior probabilities. The phylogeny was reconstructed based on nucleotides of 13 mitochondrial PCGs and 2 mitochondrial rRNA genes using Bayesian inference (BI) analysis.

## Data Availability

The genome sequence data that support the findings of this study are openly available in GenBank of NCBI at [https://www.ncbi.nlm.nih.gov] (https://www.ncbi.nlm.nih.gov) under the accession no. MW476927. The associated BioProject, SRA, and Bio-Sample numbers are PRJNA700806, SRR13664529, and SAMN17839477, respectively.
